# Vital Pulp Therapy of Young First Permanent Molars: A Retrospective Study on Radiographic Findings 24 Months Post-treatment

**DOI:** 10.3290/j.ohpd.b4586789

**Published:** 2023-11-02

**Authors:** Konstantina Chatzidimitriou, Kyriaki Seremidi, Maria G. Balta, Victoria Katechi, Konstantina Petroleka, Sotiria Gizani

**Affiliations:** a PhD Student, Department of Paediatric Dentistry, School of Dentistry, National and Kapodistrian University of Athens, Greece. Conception and design, acquisition, analysis and interpretation of the data.; b Paediatric Dentist, Department of Paediatric Dentistry, School of Dentistry, National and Kapodistrian University of Athens, Greece. Conception and design, acquisition, analysis and interpretation of the data.; c PhD Student, Department of Oral Biology and Department of Periodontology, University of Oslo, Norway. Data analysis.; d Postgraduate Student, Department of Paediatric Dentistry, School of Dentistry, National and Kapodistrian University of Athens, Greece. Data acquisition.; e Dentist, Department of Paediatric Dentistry, School of Dentistry, National and Kapodistrian University of Athens, Greece. Data acquisition.; f Associate Professor and Head of the Department of Paediatric Dentistry, School of Dentistry, National and Kapodistrian University of Athens, Greece. Conception and design, interpretation of data.; All authors drafted the manuscript, revised it critically for important intellectual content, and approved the final version to be published. All agreed to be accountable for all aspects of the work by ensuring that questions related to the accuracy or integrity of any part of the work were appropriately investigated and resolved.

**Keywords:** vital pulp therapy, pulp capping, pulpotomy, radiographic success, root development

## Abstract

**Purpose:**

With success rates comparable to that of root canal treatment, vital pulp therapy (VPT) has gained clinical interest and has been used in the management of young permanent teeth with inflamed pulps. The aim of the present study was to retrospectively evaluate the radiographic success of VPT in young first permanent molars 24 months post-treatment and correlate findings with tooth and treatment-related characteristics.

**Materials and Methods:**

Dental records of all patients with first permanent molars which received VPT in the Department of Paediatric Dentistry (National and Kapodistrian University of Athens) were retrieved. Demographic characteristics and data regarding the treatment performed were recorded. Patients’ radiographs were evaluated at 6, 12 and 24 months post-treatment by two qualified paediatric dentists blinded regarding the treatment performed. Radiographic success, reasons for failure and continuation of root development were evaluated. Differences were tested using the Χ^2^ and Student’s t-test, and possible correlations were determined by calculating the odds ratio.

**Results:**

Overall radiographic success rate at 24 months was 77%, ranging between 50% for direct pulp capping and 92% for full pulpotomy. Differences were not statistically significant. Continuation of root development was recorded in almost 1/3 of the teeth and completion in almost 1/5. No statistically significant association was recorded between the outcome and any tooth and treatment-related variables.

**Conclusion:**

VPT seems to be a reliable option in the long term for the treatment of deep carious lesions in young permanent molars.

Dental caries still remains one of the greatest challenges in both primary and secondary dentition, with the global prevalence in first permanent molars (FPM) being 29%, and more than half of children experiencing caries in at least two FPM.^1^ The percentage increases during tooth eruption, since their occlusal surface is covered by gingival tissue, allowing microbial plaque accumulation in the area.^6^ Additionally, the enamel is not completely mineralised, further reducing the teeth’s resistance to acids produced by the metabolism of fermentable carbohydrates.^8^

Treatment of caries aims at removing caries-affected tissue and protecting the remaining tissue in order to avoid any further bacterial infection.^44^ A bacteria-free environment allows the pulp to heal and deposit further dentin, whereas the presence of bacteria compromises the pulp’s ability to overcome the infection and enable further growth.^16^ The ultimate goal is to preserve pulp vitality and, in young permanent teeth (whose root formation has not been completed), promote continuation of root development.^19^ A vital pulp is considered to be a “pulp with signs of reversible inflammation or in the initial stages of irreversible pulpitis only in the area of the coronal pulp associated with the carious lesions”.^12^

For over five decades, a wide range of biologically active materials have been available to promote healing of the pulp and consequently preserve pulp vitality through minimally invasive endodontic techniques. These techniques, collectively known as vital pulp therapy (VPT), remove only infected tissue from the cavity walls, allowing remineralization of affected dentin in the pulpal wall, while decreasing potential exposure of the pulp.^9,26^ They are based on the findings of histological studies which showed that inflammation is confined to a limited area of the pulp within the exposure site, and it is not uncommon to find normal histological architecture in the coronal pulp away from caries as well as in the roots.^4,35^

With a 5-year success rate comparable to that of root canal treatment,^2,11,29,33^ VPT has gained interest and has been used in the management of young permanent teeth with inflamed pulps.^32,36,45^ Immature permanent teeth with open root apices have a rich blood supply, and their pulps are unaffected by age changes and therefore react better to such techniques. However, there is always the challenge of sufficiently removing infected carious dentin to enable healing and protection of the pulp, which in turn promotes further development of the root.

In 2019, the European Society of Endodontology issued a position statement recommending that deeply carious teeth with asymptomatic carious pulp exposures could benefit from such a conservative treatment, with a favourable outcome depending on careful preoperative assessment and good case selection.^38^ The literature shows that the success of VPT highly depends on i) the remaining pulp being either non-inflamed or capable of healing; ii) the proper control of hemorrhage; iii) the application of a biocompatible/regenerative capping material; and iv) the presence of a bacteria-tight seal.^3,39^ At the same time, treatment-related factors, such as aseptic conditions, adequate removal of infected dentin and the size of the pulp exposure, are strongly associated with the VPT outcome.^39^

To date, the evidence regarding pulp-related techniques and survival rates of materials in permanent teeth is limited and even more scarce for immature FPM. Results from the most recent systematic reviews indicate an overall success rate for VPTs that ranges between 73% to 99%, with no evidence for the superiority of a particular technique or a material used.^10,15,17,39^ At the same time, there is a lack of clinical trials with long-term follow-ups reporting on the effect of the root’s developmental stage and other treatment-related factors on the treatment outcome.^22,34^ Therefore, there is insufficient evidence to support the impact of these techniques on the long-term survival of the teeth and the stability of the developing occlusion.

Therefore, the main aim of the present study was to calculate the two-year radiographic success rate of different VPTs in first permanent molars with deep caries and signs of reversible pulpitis. A further objective was to determine whether success is correlated with various tooth- and treatment-related characteristics in order to determine possible risk factors for technique failure.

## Materials and Methods

This retrospective study reported on the 24-month radiographic success of vital pulp therapy of young first permanent molars. The study was performed in accordance with the ethical standards of the Declaration of Helsinki (WMA 2013), and the research protocol was approved by the Ethics Committee of the School of Dentistry, National & Kapodistrian University of Athens (NKUA), Greece (N532, approved on 04.10.2022).

### Sample

The sample derived from patients at the post-graduate clinic of the Department of Paediatric Dentistry, School of Dentistry, NKUA. It was considered as the most convenient sample and consisted of all patients who had been treated in the above Department between 2016 and 2021. Patients were considered eligible according to the following inclusion criteria: aged 6-13 years, with a non-contributory medical history, full dental records, first permanent molars treated with vital pulp therapy, follow-ups up to at least 24 months post-treatment.

Patients with incomplete dental records or missing radiographic documentation during the follow-ups and patients with follow-ups < 24 months were excluded from the study.

### Data Collection

Patients’ electronic records were retrieved, and digital radiographs taken at 6-, 12- and 24-month recall appointments (according to the guidelines of the European Society of Endodontics, 2019) were evaluated. The data recorded included: a. demographic characteristics (gender, age); b. tooth and treatment related characteristics: tooth number, dental arch, root developmental stage, type of VPT performed, material used and final restoration; c. success/failure, cause for treatment failure; d. and continuation/completion of root formation. Standardised forms were used to record the findings.

All evaluations were performed independently by two qualified paediatric dentists blinded regarding patients’ and treatment characteristics. The examiners were trained in the evaluation criteria prior to the study and were calibrated by evaluating 40 random radiographs not included in the study sample. To determine the reliability of the evaluations, Kappa scores were determined by the 2 examiners re-evaluating all cases 1 week after the initial evaluation. The intra-rater and inter-rater reliabilities were 0.91 and 0.87, respectively. When there was a disagreement, both examiners discussed the radiographic findings to achieve a consensus.

The datasets used and/or analysed during the current study are available from the corresponding author upon reasonable request.

### Assessment Criteria of Success

A treatment outcome was considered successful in cases with no widening of the periodontal ligament, loss of lamina dura, internal/external root resorption, and periapical/furcal radiolucency.^39^ Teeth with pulp-canal obliteration were considered successful cases, as were cases where continuation of root development or complete apical closure (apexogenesis) was evident.

### Statistical Analysis

Descriptive statistics were used to analyse initial data regarding treatment and tooth-related characteristics in each type of VPT. Success rates, reasons for failure and the effect on root development were calculated. Significance of calculated differences was tested using the Χ^2^ test and Student’s t-test. Odds ratio (OR) and 95% confidence interval (CI) were calculated to identify the relationship between the treatment outcomes and tooth- and treatment-related factors. All data analyses were performed using SPSS Version 21.0 (SPSS; Chicago, IL, USA). and the statistical significance was set at p < 0.05.

## Results

The medical and dental records of all patients treated in the Postgraduate Clinic of the Paediatric Dentistry Department, showed that 26 patients (14 males and 12 females) with a mean chronological age of 8.47 years met the inclusion criteria. Overall, thirty FPMs were included in the study.

### Sample Characteristics

Of the thirty FPM treated, fourteen (47%) were located in the upper arch and sixteen (53%) in the lower, with the majority (n = 23, 77%) being on the right side of the mouth in either arch. Regarding root developmental stage, fourteen FPM (47%) had almost complete roots with open foramen; in nine FPM (30%) roots were 2/3 formed, and seven (23%) had completed roots with closed apices. Of the VPT performed, six teeth (20%) had undergone direct pulp capping (DPC), eleven (37%) partial pulpotomy (PP) and thirteen (43%) full pulpotomy (FP). Regardless of the type of VPT, eleven teeth (37%) had been treated with Biodentine (BD) and nineteen (63%) with mineral trioxide aggregates (MTA). The final restoration chosen was stainless steel crowns for the majority of the teeth (n = 21, 70%), while the rest were restored with composite resin. Table 1 shows the distribution of the above characteristics according to the VPT performed.

**Table 1:** Distribution of tooth- and treatment-related characteristics according to VPT performed

Specific characteristics	Direct pulp capping	Partial pulpotomy	Full pulpotomy
	N (%)	N (%)	N (%)
Dental arch			
Upper Lower	2 (33) 4 (67)	8 (73) 3 (27)	4 (31) 9 (69)
Side			
Left Right	2 (33) 4 (67)	2 (18) 9 (82)	3 (23) 10 (77)
Root developmental stage			
Complete up to 2/3 Almost complete (open apex) Complete	1 (17) 2 (33) 3 (50)	3 (27) 5 (46) 3 (27)	5 (38) 7 (54) 1 (8)
Medication			
Biodentine Mineral Trioxide Aggregate	4 (67) 2 (33)	5 (46) 6 (54)	2 (15) 11 (85)
Final restoration			
Composite resin Stainless steel crown	3 (50) 3 (50)	3 (27) 8 (73)	3 (23) 10 (77)

### Treatment Outcomes

The mean radiographic success rated for VPT was 77% at 24 months post-treatment, ranging between 50% for DPC and 92% for FP (Table 2). It was evident that for all treatments, success rates dropped with time, with the lowest rates recorded at 24 months post-treatment. None of the calculated differences between the radiographic success rate of different VPTs or at different time intervals were statistically significant (p = 0.35 for 6 months, p = 0.28 for 12 months, p = 0.09 for 24 months).

**Table 2:** Success rates of vital pulp therapy at different time intervals

		Direct pulp capping	Partial pulpotomy	Full pulpotomy	Overall
**6 months**					
	Success	5 (83%)	11 (100%)	13 (100%)	29 (97%)
	Failure	1 (17%)	0 (0%)	0 (0%)	1 (3%)
**12 months**					
	Success	5 (83%)	11 (100%)	13 (100%)	27 (90%)
	Failure	1 (17%)	0 (0%)	0 (0%)	3 (10%)
**24 months**					
	Success	3 (50%)	8 (73%)	12 (92%)	23 (77%)
	Failure	3 (50%)	3 (27%)	1 (8%)	7 (23%)

**Fig 1:**
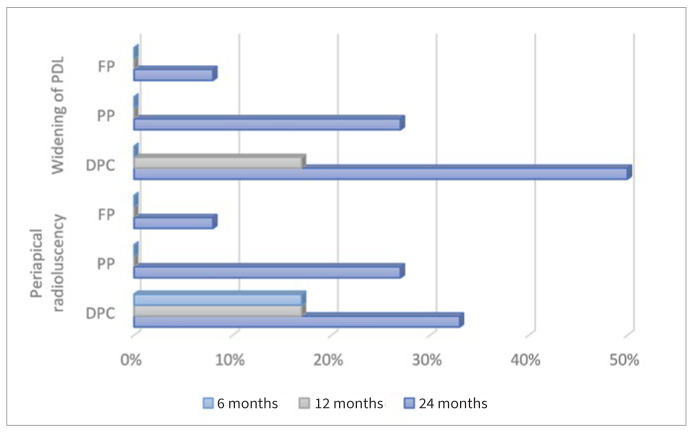
Distribution of main reasons for failure according to vital pulp therapy performed (direct pulp capping [DPC], partial pulpotomy [PP], full pulpotomy [FP]) in all follow-ups.

The main radiographic characteristics of teeth that were classified as failures comprised widening of the periodontal ligament and periapical radiolucency (Fig 1). Widening of the periodontal ligament was recorded in half of the cases of teeth treated with DPC at 24 months, with the corresponding percentages for PP and FP being 27% and 8%, respectively. However, the recorded differences were not statistically significant (p = 0.13 and p = 0.35, respectively). Non-significant differences (p = 0.12) were also recorded for all cases with periapical radiolucency, which was estimated to range from 17% at 6 and 12 months post-treatment to 33% at 24 months post-treatment for DPC. For pulpotomy, periapical radiolucency was only recorded at the 24 month follow-up, with a percentage that varied from 8% for FP to 27% for PP.

### Effect of Different Treatments on Root Development

**Fig 2:**
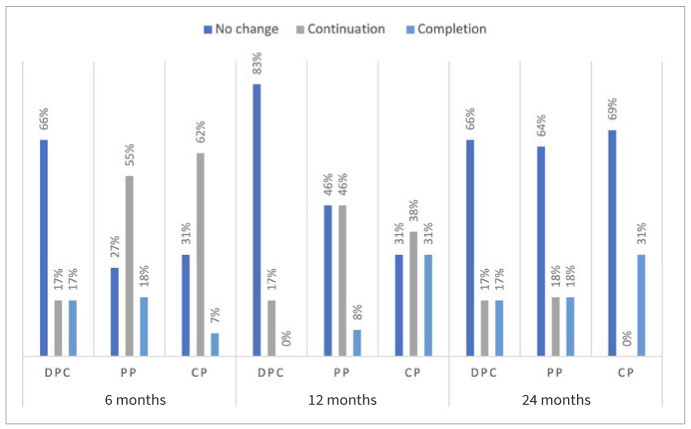
Distribution of the effect of vital pulp therapies (direct pulp capping [DPC], partial pulpotomy [PP], full pulpotomy [FP]) on root development.

Figure 2 presents the distribution of continuation and completion of root development of the teeth according to the type of treatment. Overall, 45% of all cases showed continuation and 14% completion of root development at 6 months post-treatment. Corresponding values for 12 and 24 months were 34% and 13% for continuation of root development and 12% and 22% for completion of root development, respectively. At 6 months, continuation of root development was recorded in 62% of FPM treated with FP and root completion in 18% of FPM treated with PP. At 12 and 24 months, continuation of root development was recorded in 46% and 18% of FPM treated with PP, respectively. Completion of root development was recorded in 31% of FPM treated with FP at 12 and 24 months post-treatment.

No statistically significant difference was found for continuation and completion of root development among the different VPTs at 6 months (p = 0.18 and p = 0.93), 12 months (p = 0.49 and p = 0.17) and 24 months post-treatment (p = 0.28 and p = 0.7). These differences remained statistically non-significant when the rates were adjusted for specific characteristics, i.e., age, gender, jaw and side or medication used.

### Correlation of Tooth and Treatment-related Characteristics with Radiographic Success

The final model for variables statistically significantly associated with treatment outcomes of VPT is shown in Table 3. According to the odds ratio, there was no statistically significant association between any of the tooth- and treatment-related variables with a successful outcome of any of the VPTs performed.

**Table 3:** Odds ratio (confidence interval) for the effect of different tooth- and treatment-related characteristics on the 24-month radiographic success

	Direct pulp capping			Partial pulpotomy			Full pulpotomy		
	Odds ratio	95% CI	p-value	Odds ratio	95% CI	p-value	Odds ratio	95% CI	p-value
Gender									
Female Male	2 Ref	0.33–11.9	0.5	1.3 Ref	0.46–3.8	0.58	1.7 Ref	1.06–2.77	0.62
Age in years									
< 8.47 >8.48	2 Ref	0.33–11.97	0.5	2.7 Ref	0.63–11.3	0.28	1.2 Ref	0.93–1.55	0.85
Jaw									
Mandible Maxilla	1 Ref	0.1–9.6	0.8	1.6 Ref	0.94–2.73	0.34	1.5 Ref	1.01–2.24	0.69
Side									
Right Left	1 Ref	0.1–9.6	0.8	1.3 Ref	0.89–1.99	0.51	1.85 Ref	0.96–1.85	0.77
Medication									
Mineral trioxide aggregate Biodentine	1 Ref	0.32–3.1	0.8	2.7 Ref	1.1–6.5	0.12	1.2 Ref	0.93–1.55	0.83
Root developmental stage									
Open Closed	2 Ref	0.33–11.9	0.5	1.7 Ref	0.56–1.39	0.15	1.1 Ref	0.92–1.3	0.92
Restoration									
Stainless steel crowns Composite resin	1 Ref	0.32–3.01	0.8	1.5 Ref	0.08–26.86	0.66	1.33 Ref	0.96–1.85	0.77

## Discussion

The present study, comprising data of 30 FPMs, aimed to assess the 2-year radiographic outcomes of different VPTs in first permanent molars with deep caries and signs of reversible pulpitis. Despite the limitations of its design, its long-term follow-up nature and the strict inclusion criteria increase the strength of the conclusions drawn. Results showed that success rates for all VPTs ranged from 97% at 6 months to 77% at 24 months. Regardless of the type of VPT, almost half of the teeth showed continuation of root development and one-fifth completion of root development as early as 6 months post-treatment. None of the factors related to the teeth or the treatment seemed to directly affect the outcome.

Despite the high radiographic success rates, they decreased over time for all VPTs, with the lowest rates being recorded at 24 months post-treatment. No statistically significant differences were recorded in the success rates between various follow-up intervals (p = 0.35 for 6 months, p = 0.28 for 12 months p = 0.09 for 24 months). This agrees with the results of a recent systematic review, in which both clinical and radiographic success rates of various VPTs in immature permanent molars did not show statistically differences among the 3 follow-up periods evaluated (6 months, 1 year, and 2 years).^39^ Other studies provide supporting evidence that most of the failures occur within the first months up to 1 year after VPT,^13,18,20^ and that early failures are associated with an acute inflammatory process caused by persistent bacteria, with severe pain shortly after the treatment procedure. Late failures, on the other hand, have been attributed to pulp necrosis and periapical pathology with or without bacterial involvement.^37,46^

Regarding the effect of different VPTs on root development, our results showed that regardless of the type of VPT, at 6, 12 and 24 months, 14%, 12% and 22% of the cases showed completion of root development, respectively. However, no statistically significant difference was found among the different VPTs at different time intervals (p = 0.93 for 6 months, p = 0.17 for 12 months, p = 0.7 for 24 months). Tozar and Almaz^41^ observed that 46% of the teeth had completed root development and 41% exhibited root length increase at 12 months post-treatment with partial pulpotomy. The systematic review by Tong et al^39^ reported that completion of root development was achieved in more than 83% of the cases, regardless of VPT technique used, a percentage much higher compared to that observed in our study. However, the follow-up timepoint varied among the studies included. Taking into consideration that it takes 2–4 years for complete root development in permanent molars,^24^ a review time of 2 years may not be adequate for root completion in some teeth.

In the present study, patient- and tooth-related factors (e.g., gender and position of the tooth in the arch) did not influence the outcome, which is in accordance with previous studies.^20,23,28,31^ Although there is evidence in the literature that the patient’s age affects treatment prognosis, different studies reach different conclusions, with some studies reporting that younger age is associated with a high healing potential of pulp tissue, and others failing to correlate lower success rates with increasing age.^42^ In this study, the effect of age on the success or failure of VPT was not statistically significant, a fact that can be mainly attributed to the narrow age range of the patients in the sample.

This is further supported by the fact that the present results failed to demonstrate a direct association between root developmental stage and VPT outcome. In the literature, the differences between the success rates of VPTs in teeth with open or closed apices were not definite; however, a better prognosis was observed in teeth with open apices.^5^ It is known that the dental pulp of teeth with open apices is richer in cellular structure, so the regeneration and healing potential are high after the removal of the infected pulp tissue.^27^

The materials used for VPT in the present study exhibited no statistically significant influence on treatment outcomes. A recent systematic review reported that the superiority of different materials was still inconclusive and that both MTA and calcium hydroxide provide satisfactory outcomes when used as pulp capping agents in VPT.^39^ However, VPT has become a modern treatment modality thanks to the development of calcium silicate cements.^7^ Although calcium hydroxide-based materials have historically been used for various VPT treatments,^21,33^ due to their disadvantages, contemporary hydraulic calcium silicate cements are the biomaterial of choice for VPT because of their favourable sealing, antibacterial action, and excellent biocompatibility.^25,40^

In terms of restoring teeth following VPT, it is now accepted that immediate restoration of the teeth following VPT providing a good coronal seal plays an important role in treatment success.^20,38^ In general, large direct composite resin restorations may have a short life span,^7^ while restorations with stainless steel crowns (SSCs) have been considered superior by providing better crown integrity and lower risk of restoration or tooth fracture.^14^ In our study, all restorations were performed immediately following VPT and the majority (70%) of the molars were restored with SSCs. Therefore, the type of restoration did not statistically significantly affect the 24-month radiographic success of VPT; this can be attributed to the high sealing ability and the low annual failure rate of SSCs, which is supported by previous studies on dental materials.^1^ Özgür et al^30^ reported only marginal discolouration of the final resin restoration in pulpotomised immature FPMs, without any signs of clinical or radiographic failure.

### Limitations

The present study was subject to the inherent limitations of any retrospective clinical investigation. Apart from the sample being the most convenient, specific operator-related factors were not as consistent as in prospective trials with calibrated operators. Moreover, the quality of the database was based on accurate record keeping and complete documentation, limiting the sample as cases with incomplete dental records or missing radiographic documentation during the follow-ups or patients with follow-ups < 24 months had to be excluded. Τhis justifies the small sample size gathered in the present study despite the inclusion and exclusion criteria applied being strict. The restricted follow-up period of 24 months was another important limitation of the study. However, data from a systematic review suggest that 6 months or more may be considered as a suitable follow-up period to evaluate the success after a partial pulpotomy.^15^ Nonetheless, studies with a more extended follow-up period and larger sample size are necessary to assess the long-term treatment outcome of VPT.

## Conclusions

Within the limitations of the present study, DPC performed equally well with partial and full pulpotomy at 6 and 12 months. At 24 months, full pulpotomy showed the highest success rate, although no significant difference was found with the other techniques. Continuation of the root development was more prominent, but not significantly, in cases of partial and complete pulpotomy at 6 and 12 months as compared to DPC. Although, none of the patient and tooth related factors were significantly associated to the outcome, the choice of the appropriate technique of VPT based on dental history parameters as well as clinical and radiographic findings seem to be of outmost importance.

### Why is this paper important for paediatric dentists

Nowadays, vital pulp therapy has started to gain interest in the field of paediatric dentistry and its use has been increased for the management of young permanent teeth. However, up to date there is limited evidence regarding its long-term success.

Based on the 24-month radiographic findings of the present retrospective study, VPT seemed to be a reliable and promising treatment approach for the management of FPM. Given its high success rate, VPT should be considered by paediatric dentists as an alternative treatment option to endodontic treatment.
